# Southern Dental Association

**Published:** 1886-11

**Authors:** 


### PROCEEDINGS OF SOCIETIES.

### Southern Dental Association.

EIGHTEENTH ANNUAL MEETING.

NASHVILLE, TENNESSEE, July, 27th to 30th, 1886.

(Reported for DENTAL REGISTER by "Mrs. M. W. J.")

The Eighteenth Annual Meeting of the Southern Dental Association was held in Watkin's Institute, Nashville, Tennessee. Opened with prayer by Rev. J. D. Barbee.

President W. C. Wardlaw in the chair. The minutes of the seventeenth annual meeting, held in New Orleans, were read and confirmed.

Dr. J. H. Prewitt delivered the following:

### ADDRESS OF WELCOME.

Gentlemen of the Southern Dental Association: It has been made my pleasant duty to welcome you in the name of the Dental Association of the State of Tennessee. First, I desire to acknowledge the honor conferred upon me and my inability to fitly convey to you by words the sincerity of that welcome. I am happy to know, however, that there are to you other evidences of your welcome than the words of the representative of the State Association.

While it has never been my pleasure, nor that of a number of gentlemen present, to meet with you in Association capacity, yet you are no strangers to us. As a body of investigators and as an educator in so far as Associations are educators, we regard you as second to none, though it may be less pretentious, possessing merit without assumption, seeking to spend and be spent in the upbuilding and advancement of that profession which, next to the wife and little ones, you love most.

Upon your rolls are the names of those whose reputations are not confined to any section, but whose names are familiar to the profession in every clime.

There are those here who were at the forming of this Association still active and aggressive. Some who took passage when the ship was full rigged, and some who did, yesterday as it were, enlist among you. Still there are others standing ready with credentials regularly made out, and waiting impatiently for permission to be numbered with you. To all we say, you are welcome.

True it is that you have all doubtless expected an address of welcome. Whether you were really so or not, in these piping times of peace, when all seems going as merry as a marriage bell, it is but a part of every programme; it is taken as a matter of course, that upon all occasions like the present some gentlemen will, with glittering speech and well-rounded sentences after the fashion of form, say, "you are welcome."

We would make you know beforehand, if language were not so poor, what you can know only when you are gone. You come well, in that your coming promises not only a flow of soul but a feast of reason.

You have lived, may be, for months in happy anticipation of this pleasant re-union. When you would strike hands with your brothers, and enjoy again that social intercourse which these meetings invariably develop. Nor is that all; you have been stowing away incidents of interest for this meeting, and have been plying with all your might the principles of science to every branch of the profession that you might present matters of interest for the consideration of this body. The strides made by the profession in the last quarter of a century are giant ones, but this is no argument that there are not yet many such to make, and to you the profession as well as the people of your section especially, are looking as a body of representative men to perfect as far as possible methods, theory, and practice. I forgot, however, that it is no part of my business to direct or even forecast the deliberations of this body, but only to tell you if I can how welcome you are.

In that you are in some measure the guests of the Dental Association of the State of Tennessee, you are welcome. In that you come a body of representative men of the profession, by dint of study, application, and experience, prepared to discuss and demonstrate from every standpoint matters of greatest interest to the dentist and his patient, you come well.

We rejoice that while this word of greeting has been made to do duty under so many varied circumstances, yet on no occasion, has it been intended to be more heartily employed. Except when used in hollow form no word, perhaps, expresses more. None but the brave officer who cried in the agony of his soul when the fortunes of Waterloo were trembling in the balance, "Would to God, night or Blucher would come," can know how welcome either would have been.

When Cæsar had received from Brutus' arm, nerved by ingratitude, the fatal stab, he ceased to struggle, and with the words "et tu mi filli," he wrapped himself in the more than royal robes of Rome reeled and fell at the foot of Pompey's statue "welcoming death."

Not till the Peri had brought a sigh of repentance were the gates of heaven thrown wide open and he "welcomed" in. Expressive, beautiful, comprehensive, take it in its most expressive sense and it is thus we mean it. Make the most beautiful application of it and it is thus we would apply it. Make the broadest and most comprehensive use of it and you have used it as we would.

While we welcome you we know it is to work, as well as pleasure, and it may be well for each one to ask himself the question:

"Why all this toil for triumphs of an hour?
Life's a short summer, and man's a flower.
By turns we catch the vital breath and die,
The cradle and the grave, alas, so nigh!

Why am I here? for what? Is it that this meeting may better prepare and fit me to serve my patients? Have I in view the betterment of the condition of my fellow man? Is it that by contact with the better element of the profession my views may be broadened, my capabilities enlarged, my zeal increased? If so, then there should be no drones in this hive, but each one should contribute his mite, and he who feels himself not a good talker, or has nothing to say, let him see to it that he proves himself a good listener, and that he watch as well pray.

In conclusion, and in view of the very sacred and intimate relations existing between the dentist and his patient, and in view of the further fact that in the profession since this body last met, death has dealt his shafts both right and left, and whole battalions lie a-field, it may not be out of place to admonish and remind one another to--

Make, then, while yet ye may, your God your friend,
Whom Christians worship, yet not comprehend;
The trust that's given, guard, and to yourselves be just.
For, live we how we may, die we must.

Again we say, you are welcome.

To Dr. Prewitt's address, the following response was made by Dr. Geo. H. Winkler:

*Ladies and Gentlemen and Members of the Tennessee State Dental Society:* I am happy in being permitted as the humble mouth-piece of the Southern Dental Association to return our thanks for the hearty welcome you have just extended us to Nashville through your eloquent orator, Dr Prewitt. We appreciate most profoundly the kind sentiment you have expressed, the warm greeting you have tendered. We believe the unquestioned generosity which actuates you in extending your hospitality is only equaled by the whole-souled confidence and pleasure we feel in accepting it. We are delighted to be in Nashville to-day. Nashville—city, port of entry, capital of Tennessee. When we look around and mark the evidences of greatness displayed by your people we feel that we are indeed in an advanced civilization; a civilization encompassed and maintained by men of great energy, ability, integrity, business accomplishments and God-fearing virtues; men whose hands have certainly been upheld in every glorious and virtuous undertaking by women clothed in the purest of all womanly attributes. Your commerce is borne upon the bosom of the Cumberland; your traffic is carried by railroad to every section of your country. We note your business houses, your mills, and your factories, your foundries and machine shops. We are lost in admiration of your magnificent state-house, from whose lofty dome the grandest view of hills and valleys, of river and mountain, is stretched out before us in the most beautiful panorama of nature known in the United States. Your public buildings are elegant, your private residences are beautiful, your institutes of learning are powerful factors in the civilization and progress of our country; they are emphasized at home in stone and are held in high repute abroad through the ability of their alumni. Your dental schools are unsurpassed in their appointments, and are presided over by able and learned instructors; their graduates bear testimony to thoroughness of preparation for their life work, both by the professional reputation they enjoy at home and the high standing they hold among their fellows. We congratulate you, gentlemen, upon the successful establishment of a department of dentistry where our colored brother who desires to practice dentistry can be prepared. Some of the colored people will practice dentistry, and it is good that they are now provided with a school where they can be properly educated, and we feel glad that the only institution where they can be made useful and honorable has been established in this southern city. Your eleemosynary institutions are numerous and complete. Conspicious among them are the asylums built by loving hearts and willing hands for taking care of, raising to useful and virtuous lives those unfortunate little ones whom cruel destiny has forced in this world to look for help to friends and for parental guidance to God. Just beyond your confines your philanthropy has reared a home for those of your people who are sorely afflicted through that most terrible of all maladies, the dethronement of reason. Your daily, weekly, and monthly publications are models of literature, and one of your publishing houses, sending its works over the whole world, disseminating its God-given truths to the uttermost quarters of the earth, fills with pride our American hearts. The great number of .churches whose spires and domes here point to heaven proclaim you a God-fearing people, and the pride and pleasure with which you point to the "Hermitage," situated almost within the limits of your city, the home of the great and valorous Andrew Jackson, bespeak your patriotism.

We are proud to be with you to-day, gentlemen. We are happy in a true appreciation of the honor of your welcome. The pleasant, auspicious opening of this session of our Association inspires me to predict a glorious meeting. The occasion is propitious and we are determined to exert every energy and every honorable means to make it memorable in the annals of our Society. Our friend, Dr. Prewitt, in quoting poetry has brought to mind a stanza--

"Ah, that sharp moment, when before our eyes
The glowing metal on the anvil lies,
Comes to us, not once, but day by day,
To bless or curse us as it slips away.
Its strenuous summons calls us as we wait,
'Strike while the iron's hot and forge your fate,'
And wise men hear and heed; the rest are fools,
Who stare and trifle 'til the iron cools."

We see before us, gentlemen, a glowing opportunity that we do not intend to let pass us, of forging another link in the chain that binds us in fraternal intercourse, in generous variety, in honorable emulation.

We again thank you for the delightful welcome, and promise to exert every honorable means and the most indefatigable energy to make this meeting a grand success and cause it to redound to the best interests of our Association, and to the honor and dignity of our cherished profession.

### Dr. Thackston's Address.

Immediately following Dr. Winkler's address, Dr. Thackston, of Farmville, Va., gave a hearty invitation to the Association in the following words:

Fourteen years ago upon the banks of the "James," in a city sitting upon her "seven hills" and looking mournfully down upon her battered walls and ramparts, upon her crumbling ruins, and upon the graves and monuments of her illustrious dead, it became my duty, as it was my honor and pleasure, to welcome you to all that was left of that grand old city, and to all that remained of that grander "Old Dominion"—to her wasted homes; to her broken altars; to her humble hearth-stones and to her hospitable roof-trees; but amid all her wreck and ruin, to hands as clean, to hearts as warm, and love as pure as bannered knight or crested cavalier e'er dared maintain in "tented field" or glittering tourney.

The years have come and gone, and gone as to some whose hands we then struck and clasped in the fond hope that we should meet again. And some who faithfully shared your every burden, who rejoiced in all your triumphs, and who justly and worthily wore your proudest honors, now and here, under the dispensation of a benignant Providence, I meet again, upon the banks of another beautiful river, in a young, a populous, and flourishing city, instinct with life and energy, busy and prosperous, teeming with commerce and traffic, decked and crowned with marts of trade, with temples of art, with temples of science, and temples of worship. And here in this honored and august presence, amid these grand and imposing surroundings, in this hall, I bring you once more the greetings, the salutations, and all-hail of that same "old mother," who, still regarding you as in large degree her own social, political, and professional offspring, repeats the proud exclamation of the Roman matron when pointing to her children: "These, these are my jewels."

And, now, what shall I say in acknowledgment of the reception you have accorded me and my honored colleagues? What offering can I lay upon your altar worthy your acceptance and commensurate with your kindness? It would be vain and presumptuous to essay the role of teacher, for you are adepts and experts in art and proficients and scholars in science. You are not only my peers but my masters. I bring you no new "dispensation" and can unfold for you no "late revelation." I have solved no hidden problem and can make no grand and imposing contribution to your stores of knowledge. Why, then, you may ask, am I here? And I will answer: To meet you face to face; to look into your hearts and to show you my own; to strike hands and touch elbows; to labor with and learn of you, for you can tell me that I never heard and teach me that I never knew.

I am here with my honored colleagues as the joint bearer of the salutations and benisons of our "State Association;" here not only to greet you fraternally but to tell you that we feel pride in this federated organization, which is the legitimate outcome of the "State Associations" that Virginia was the first to establish and incorporate, to say that we recognize and honor you as our "upper house," as the dental senate of the South, and also to fling wide open our gates, our hearts and homes and ask that you will once more honor the "Old Dominion" as your place of annual assemblage.

And in addition to this delegated and representative duty, there were personal considerations and attractions that irresistibly drew me hither. I desired once more to take counsel and compare notes with old friends and to meet and make friends of the young and rising representatives of our profession in the South and West—the men around whom cluster the fond and proud hopes of that bright and promising future to which our hearts and eyes are turned. I desired to commune, and possibly for the last time on earth, with some of those who in "days lang syne" trod with me the stony paths of our "wilderness"—who tasted with me the "bitter waters" of our professional "march;" and notably of these Dr. W. H. Morgan, the honored "Dean of Vanderbilt," who with strong arm, with dauntless courage, and with abiding faith for forty years has "smitten the rock," and who now beholds flowing around him the sparkling crystal streams of a science no longer discredited and repudiated, but recognized, respected, honored, and taught in our "groves and academies," our colleges and universities, and more than that, practiced with a profit and beneficence to society that distinguishes no other era in our professional history. Thank God, this grand old man has been spared to see the seed he helped to plant in this virgin soil germinate, grow and bud and blossom, and at last bear fruitage to his labors, and we can only say, may he "rest under the shade," and his declining years be sheltered, fanned, and refreshed by the foliage he has so faithfully nurtured, and pruned and trained.

Another strong attraction, Mr. President, was your corps of southern and western editors and journalists, the gentlemen not of our "fourth but eminently of our first estates," the gentlemen who spread our monthly and quarterly feasts and banquets—who trim our sails, who stand at the helm and navigate our barques—they who dress our thoughts and clothe our ideas, and shed upon our pathway the meridian light of this wonderful day of progress and professional development.

For these reasons and considerations I have left my home, have deferred pressing duties and obligations to accompany my fellow-delegates and be with you to-day. And now I will only trespass upon your time and attention to again thank and tell you that I shall cherish this occasion and the reception you have accorded me as one of the ever bright and green spots in life's long journey as the fulfillment and realization of my highest aspirations and as a princely recognition of my claims to your consideration. And now in closing this reply to the knightly, courteous, and fraternal introduction I have received, I shall presume upon your kindness and beg an humble place in your ranks while we are together, and a small niche in your hearts, when my life's work is over and I have become to you and to all--a memory and a shadow.

Dr. Thackston is the oldest living dental graduate and the only survivor of the second class of the Baltimore Dental College.

The President's ANNUAL ADDRESS embodied an able discussion of the question: Is Dentistry a distinct profession or should it be regarded as as pecialty of medicine? (See page 491 of October number.)

After the preliminary business of organization, the Committee on DENTAL EDUCATION reported three papers; one from Dr. B. H. Catching, of Atlanta, Ga. (See page 537 of this number); one from Dr. W. D. Dunlap, Selma, Ala., and one from Dr. M. C. Marshall, Little Rock, Ark.

These papers, though bearing the same title, treated the subject from different standpoints.

*Dr. Catching* urged the necessity of doing away with the present system of separate dental education and the separate degree, educating all dentists first as physicians—the degree of M.D. to be supplemented by a thorough education in practical dentistry. In this way only dentistry will attain the plane on which it will command the highest respect of the people, and fill its full sphere of usefulness.

*Dr. Dunlap* commended the system of private instruction under a competent preceptor, in connection with the present system of college education; learning the use of tools, and training the hand and eye while carrying out the ideas of his master. He thus knows his deficiencies and necessities and is prepared to enter college and prosecute his studies to much greater advantage than if he enters fresh from the field, or even from the literary school.

Dr. Marshall's paper dealt with the value of associated effort as a factor in education.

He said that since the American Dental Association was organized it has built up a literature which in the future must be, as it has been in the past, an exponent of the dentist's thoughts. Associated labor had been the cause of the success in all efforts to accomplish great things. It is a beneficial result which will make its impression everywhere. He advised the organization of State Associations, where all could exchange their views, thus making all wiser.

The President's Address, bearing upon the same general topic, was included in the discussion, which was continued through the second session.

*Dr. J. J. R. Patrick* said this was a stupendous subject, and like the harp of a thousand strings, its variations could never be exhausted.

He gave a comprehensive view of the rise and progress of the medical and dental professions, and the establishment and recognition of specialists in various branches. He paid an eloquent tribute to the memory of John Hunter, up to whose time but little advance had been made beyond compilations of what had been known from the time of the Greek and Roman empires.

Up to the time of John Hunter there was not one step made in the world toward dentistry. Up to the time of John Hunter anatomy was unknown. He first described the growth of the human teeth and the development of the human jaw.

*Dr. W. H. Morgan* said that when he began studying dentistry there were but two books. He then gave a history of dentistry from the organization of the old American Dental Society up to the present time. He said that dentistry has been looked down upon, and for a long time was denied a chair in the colleges. Dentistry has always taken care of itself, and I have a suspicion that she can continue to do so. The progress she has made has been developed by her own power. The United States is far ahead of England in dentistry, and it leads the world, based upon her good colleges.

*Dr. Catching* defended the position taken in his paper, in opposition to the views of Dr. Morgan, who, he said, viewed the question entirely from the college standpoint.

*Dr. Salomon*, in general, took Dr. Morgan's view, but demanded firstly a preliminary examination for students, secondly an extension of the course of study as well as a more thorough knowledge of medicine. He did not wish to disturb the usefulness of the dental colleges which have elevated the American dental profession above all others. "Gentlemen, do not go back on your alma mater," said Dr. Salomon. Dr. Catching, I can see in his eye, is sorry for what he has said. He certainly owes, as well as I, his professional knowledge to the dental college. His ideas and mine are identical, that, in the future, every dentist must first be an M.D.

*Dr. Teague* said that Dr. Morgan looked upon the subject from the standpoint of a professor. He is an honest and able man and Dr. Catching is equally good in his intentions, but looks at the subject in a different light. He continued, discussing the subject from both points of view. He thought that too many uneducated men were given diplomas A student should be under an educated man and the preceptor should be an educated man.

*Dr. H. J. McKellops*, of Missouri, said he was a self-made dentist. What he knew of dentistry he learned himself. The dental colleges were doing all they could do to further the interests of dentistry. Last May 2,200 doctors met in St. Louis and did not neglect dentistry. They opened their doors and invited us in. They recognize our growth. But dentistry will be a separate profession. No teaching on earth, without your own application, will enable you to make your mark in your profession. Only personal exertion will place a man on the top round of the ladder. He said there was no money in the business of college professor; it meant money out of pocket every time.

*Dr. Wright* (S. C.) thought the mechanical and operative departments should be kept entirely separate. He was glad to say that in some of the States, and especially in Georgia, through the efforts of the State Dental Association, the dental law was such that no man could now enter upon the practice of dentistry until he had proved himself competent.

*Dr. W. H. H. Thackston* spoke of the time when alliance had been sought with the medical profession. The latter having turned the cold shoulder, dentistry had been driven to depend upon her own resources. Under the present system of education a history had been made of which they had the right to feel proud, and he hoped the system would not be hastily abandoned, because, under it, some men had not turned out all that they ought to be. He said that no system whatever could force ideas through thick skulls, or fill empty heads with brains; that but few men had the natural endowments fitting them for dental cadets, though they might make respectable lawyers, doctors, or preachers. When the millenium comes, it will make little difference, but just now we should be careful not to make too wide departures from established precedents.

*Dr. Johnston* (Va.) said that with the best dental laws that could be enacted, there were many incompetent men now in practice who could not be reached. We can't educate them, but can only wait till they die off. Good as the present system of education is, something is needed; there are too many colleges, and they have not the means to employ superior talent. The only way to accomplish what is needed is through endowed universities. This is the secret of the success of the Dental College of the University of Michigan, and of Harvard; the chairs are endowed, and the best talent can be paid for.

*Dr. Crawford* said the science and art of dentistry are fascinating. The dentist rarely abandons his profession, yet lawyers in some instances, and men of other associations in life, study dentistry. It is a significant fact that it should be regarded as highly as it is, even in its present crude state. The idea that the dentist should know medicine has come to stay.

*Dr. Stubblefield* (Vanderbilt) said that though we would be glad to have only literary graduates for dental students, giving a broad foundation on which to put the capstone, yet it was necessary to do the best we could with what we have. He would not exclude a man from the profession simply because he could not pass according to the latest rules.

We all honor Dr. McKellops; if with the fine material nature gave him, he had had the educational advantages of to-day, what might he not have accomplished? Native ability highly educated, can accomplish everything. He himself had left the medical profession to become a dentist, and he had never regretted it. God had given him certain mechanical ability which found its proper outlet in dentistry. He was an advocate of higher education; education made a man more capable of exercising his mental faculties, of developing his powers, of doing better work; it gave him a good basis to work upon.

*Dr. Morgan* said he was in favor of a broader education, as far as teaching the sciences in their application to dentistry, but as far as a medical education went, he did not think there was any necessity for a dentist to understand midwifery or pharmacy; the treatment of yellow fever or of cholera. One science after another has been brought in till we are in danger of having all the *issues* and all the *pathies* which endanger and destroy life rather than preserve it. The profession at large has nothing to do with illegal diplomas, and is not responsible for the damage which they have done abroad.

After some further discussion of the same points, the Association adjourned to 9 o'clock A. M.

WEDNESDAY, July 28, 9 A. M.

The subject of education was taken up again, and Dr. W. H. Morgan introduced Dr. Hubbard, dean of the medical and dental departments of the Meharry Medical College, an institution devoted to the education of colored men for the profession of dentistry. Dr. Hubbard gave the Association a history of the college, speaking especially for the dental department which will be opened in October. He bespoke for their dental graduates the same kind reception that the medical profession had extended to their medical graduates, of whom sixty-two are now located in Southern and Southwestern States. The subject was then passed, and the report of the Committee on HYGIENE called for.

*Dr. B. H. Trague* read a paper on the

PERSONAL HYGIENE OF THE DENTIST.

He contrasted the health conditions of early practitioners, when itineracy was the rule, and dentistry was a health-giving occupation, the dentist having continual change of scene, air, and diet; physical exercise in the muscular work of hand pressure, and the rolling-mill and the blow-pipe in the laboratory, and the present sedentary life of the city dentist confined in small overheated, badly ventilated offices. Men who began practice many years ago—on the old style, are now hale, hearty old men; while the younger generation are too often pale, slender, delicate men, afflicted with various ailments of stomach, liver, lungs, and nerves. Relief from biliousness, dyspepsia, and sick headache, is sought in bitters, pepsin, and toddy, the nerves are soothed with the strong cigar, rest is sought on the lounge, while the midnight oil is burned reading journals and recording incidents, until the overstrained nerves prevent sleep, and life becomes a burden. The remedy for all this is to be found in physical exercise, on foot or on horseback, or the bycicle, or in the gymnasium, if the weather does not permit out-door exercise; this should be made a daily duty. Diet should be carefully regulated and no excesses allowed, relying on the normal stimulant of exercise and a healthy appetite; never on blue pills or liver regulators. Never rely on Lubin's Extracts to cover the odors of hourly smokes or dirty linen. Patients will learn the best operator, under these circumstances, in favor of a clean, sweet dude. Purity of person is of the greatest importance, and especially sweetness of breath.

The arm-rest should be used to support the uplifted arm, and the stool to obviate the bent body and compressed viscera and hanging head; habitual standing also gives hemmorrhoids and varicose veins. The engine should be run by an artificial *motor* of some sort, or one leg is liable to become useless from disease, the other injured by overwork. Equipoise of mind should be cultivated, guarding against worry and fret. Sleep plentifully and avoid late hours. Care of the eyes is peremptory, to this end the light must be skillfully admitted, glare avoided, neutral tints selected for the walls, etc., and the eyes occasionally examined by a skillful oculist.

Much nervousness and irritation may be avoided by systematic appointments and short sittings, giving short rests to overstrained portions, and agreeable change. Guard against breathing over the exhalations of the patient; have easy-fitting, broad, solid shoes; keep cool, and take an annual respite from work when you can have a dip in the sea or a breath of mountain air, with all this attending the Annual Associations. Then your life may be long, and filled with happiness for yourself, and usefulness to your fellow men.

This most suggestive paper was discussed at length by Drs. Winkler, Richards, Adair, J. Hall, Moore, Morgan, Chisholm, McKellops, Rembert, Wright, Wardlaw, Freeman, and Thackston. After which the Association adjourned for the exhibition of Appliances.

*Dr. McKellops* exhibited the instruments, matrices, and other appliances, as used by Dr. Herbst in his clinics.

His idea of making his barbed broaches of gold wire, which in case of breaking off, can be left in as part of the filling, doing away with the "sweating for hours" over a broken steel broach, deserves especial mention.

*Dr. Dunlap* sent some jaw-bones taken from Indian mounds, which, as pointed out by Dr. J. J. R. Patrick, showed plainly the marks of the ravages of caries, alveolar fistula, and other diseases attributed to modern civilization.

*Dr. Staples* (Texas) showed a foil folder and crumpler, or crimper, and a paper disk carrier.

*Dr. H. E. Parr* exhibited his separators, which were quite different from Perry's, but very effective.

*Dr. Morgan* showed copies of a little book entitled Dental Questions and Answers, an epitome of the Lectures of Dr. L. C. Ingersoll, and a valuable *vade mecum* for the student or the practitioner, entirely orthodox in its teachings.

*Dr. B. S. Byrnes* showed his appliances for regulating teeth, with models of cases "before and after" regulating.

At the afternoon session, Pathology and Therapeutics, Histology, and Microscopy, Chemistry, and Operative Dentistry were called before a paper was reached.

*Dr Crenshaw* read a paper entitled :

### A COMPLETE SYSTEM POSSIBLE IN FILLING TEETH.

He said that he thought a complete systen in filling teeth was possible, by putting together the principles endorsed by such leading men as Atkinson, Marshall, Webb, etc. The first principle was the restoration of the anatomical form of the tooth, which if naturally below the idea should be cut and trimmed and restored, until they touch each other in the normal way. Teeth thus restored in gold at the point of contact are safe from decay. With the use of Perry's Separators and the rubber dam, the electric mallet for driving in the gold and cohesive gold in all cases, a complete system is possible, but when the material is varied, changed, or abandoned with every variation of circumstance, systematic work is impossible. Good fillings may be made with soft foil, but as good if not better can be made in every case with cohesive gold. The advocates of soft foil claim everything for the matrix, cramming soft foil into compound cavities made simple by a matrix, but matrices are a snare and can only deceive. With the electric mallet the rapidity of blows can be regulated with exquisite pleasure, and the gold perfectly laid over floors, over margins, and against walls, even at the most critical and inaccessible points. Hand and automatic mallets are risky devices, but the stroke of the electric mallet is decisive, positive, and perfect. Only with the electric mallet and cohesive foil can uniformly perfect systematic work be done.

*Dr. Salomon* read a paper sent by Dr. Jas. S. Knapp, on

### NERVE CANALS.

The writer argued against the probabilities of success in retaining the vitality of the pulp when caries has exposed this delicate tissue. Notwithstanding the praiseworthy efforts of operators exerting their manipulative skill and the best known remedies, it is admitted as a general rule that exposure of the nerve is soon followed by loss of its vitality. The best operators now remove entirely every diseased nerve and fill the canal to the foramen. Alveolar abscess always follows a decomposing pulp left in the chamber and canal. Though in its primitive condition of health it had important functions to perform, it is idle to attempt its preservation when diseased. Its removal is more easily said than done, unless the crown is much cut away, and replaced in filling. It is needless to say that the opening into the canal must be enlarged and made funnel-shaped to facilitate the passage of instruments and the removal of contents. Using a pointed osier or orange-wood stick placed at the entrance of the canal, and tapped two or three times with the mallet, the pulp will adhere to the wood, or will have been pressed to one side, when it can be removed with a broach wrapped with cotton.

If there is no encysted sac, the tooth should be filled as soon as deprived of its contents, or if there be a sac, with an opening through the plate of bone and the gum tissue, there is no objection to filling at once. In all cases where pus originates in a sac and escapes through the cavity, it must be treated by antiseptics for a week or ten days. The canal can be filled with gold, or lead, or os artificial, or Hill's stopping dissolved in chloroform, or with wood, the last being the most easily shaped to fit the canal; it should be dipped in carbolic acid.

These papers were discussed at some length, the general voice being in favor of combining the best methods of the best operators, and selecting the materials and means best adapted to the case in hand; and also in favor of the preservation of the pulp wherever possible.

*Dr. Chisholm* announced the reception by mail, of a paper on Dental Education, from Prof. Gorgas. The subject having been passed, the paper was read by title and referred to the Committee on Publication; also a paper from Dr. Woodly, of Virginia, on Hygiene.

### HISTOLOGY AND MICROSCOPY.

*Dr. E. S. Chisholm*, of this committee, read a paper on the importance of actual demonstration as a source of evidence. He said that the first reason for histological research to the dental practitioner has obtained in the etiology of the decay of the human teeth, which implies an understanding of both physiological and pathological conditions. To know wherein consists the preventives, and the proper remedial agents for lesions already begun, an understanding of the histological conditions revealed by the microscope gives the proof of ocular demonstration. All the truths of a subject, aggregated and classified, constitute the science of that subject, but if any of the conclusions have not been subjected to actual test, it is neither science nor truth. We can demonstrate that vegetable acids break down tooth-structure, but the part that bacteria and other microscopic agents play in the destruction of the teeth has not been demonstrated. Their presence has been demonstrated, however. Demonstration is the witness, reason is the judge, verdict is the science.

The paper passed without discussion.

*Dr. R. B. Adair* (Gainsville, Ga.) was introduced by the President, as an expert in the treatment of PYORRHEA ALVEOLARIS, who has come prepared to demonstrate his method of treatment, but not having been able to obtain a patient, would take the floor.

Dr. Adair said that he had not come prepared to make a speech nor to read a paper, he had only expected to give a clinic, but not having been able to find a case for treatment, he would give them his remedy, the result of experiments begun some eight years ago. Rigg's treatment was entirely surgical, but in many cases that was only the beginning, though very essential as the beginning of treatment; but it would not restore lost tissues; the pockets are still there, and food will continue to be crowded into them, keeping up the irritation. Various attempts have been made to protect the parts, but all prove failures sometimes. With his present mode of treatment he has no failures to record, in restoring lost tissues, tightening the teeth, and bringing the gum up to its natural contour. There are two preparations, as follows:

1. Crystals of iodine are dissolved in enough pure wood cresote to make a saturated solution, which improves with age. This is applied down in the pockets and all over the suppurating surface, destroying germs and stimulating healthy granulation.

2. A glycerole of tannin is made by packing in a small wide-mouthed bottle, as much crystals of tannin as it will hold, adding enough glycerine to dissolve it, making a very thick solution. The surfaces being dried, and protected from saliva with napkins, this is applied; the overflow of saliva coming in contact with it, forms a tannate of albumen forming a pellicle which resists friction sealing up the pockets and protecting them perfectly. The patient should be instructed not to disturb this.

The next day this pellicle should be peeled off—coming off like the skin from an eggshell—and the two applications repeated. Healthy granulation takes place, and the pockets fill up with new tissue, in a period varying from ten to one hundred days, according to the severity of the case. The deposits should be thoroughly cleaned off, and the surgical operation completed at the first sitting, that the granulations may not be disturbed.

*Dr. B. H. Catching* said that he could vouch for the success of Dr. Adair's treatment, though his own was somewhat different. He believed there was always caries of the margin. If the process should first be trimmed very thoroughly, sulphuric acid should then be applied, followed by iodine if the case indicates this. Then for stimulation use chloride of zinc, forty grains to the ounce. For each patient he has a sable hair brush, with the patient's name written on it (which he burns when the patient is dismissed). Every part can be reached with the brush without causing irritation.

*Dr. G. Chisholm* said he had been very successful with Dr. Adair's treatment when patients could be induced to return for treatment. He also uses the following mouth wash:

| | |
|---|---|
| Crystals iodine | 3 grs. |
| Tincture aconite | 3 drams. |
| Myrrh | 1 oz. |
| Tannin | 10 grs. |

Alcohol sufficient to make 3 oz., flavor with wintergreen. Use on the toothbrush, without water. This keeps the mouth in good order, and prevents the formation of pus.

The main point is to remove all debris and necrosed bone, and the great difficulty is to have patients return after they are comfortable.

*Dr. Morgan* said the great principle was the removal of the cause. Unless we get at the pathology of the disease, which was not alluded to by the speakers, the treatment was purely empirical and quackish—not meaning to intimate that these gentlemen were practicing from that standpoint.

*Dr. Crawford* thought the disease might have its origin in the cementum, the pericementum being the first structure to make outcry. If it depends on constitutional conditions, we must go beyond local treatment; otherwise it is only palliative, and there will soon be a recurrence. There is nothing better for inducing healthy granulations than touching with pure carbolic acid after washing out, and drying with an absorbent.

*Dr. Thackston* (who was greeted with applause) said that in the teachings of half a century ago—which he was the only one left to represent—Horace H. Hayden, one of the fathers of American dentistry, who had given this subject years of patient investigation, called it "conjoined suppuration of the gums and alveolar process." He taught that while local, it was the local exhibition of constitutional disease; of a vitiated and impaired nutrition of the general system; his treatment embodying the building up and repair of the whole body to keep in abeyance the ravages of this disease, but there was no radical cure in his opinion. With the co-operation of intelligent careful patients, it might be abated and modified, but there was no radical cure. The success of Rigg's treatment has been far in advance of anything accomplished by Hayden or the students of that day. He still believed that he was correct as to the cause of the disease, but the thorough removal of local causes, aided much the effect of tonic treatment. He accorded all honor to the gentleman who has fallen upon a combination so wonderfully successful, but he still clung to the idea that general treatment should be combined with local--remedial, and alterative with surgical.

*Prof. J. Taft*, being repeatedly called for, took the floor.

He said that he had no extended remarks to make; though this was a subject in which all were interested, a man being no longer excusable if he has not some opinion on the question. Its nomenclature was the first point to be considered. Names sometimes became fashionable or popular, which were not appropriate to the different manifestations in different cases. "Rigg's Disease" was not an appropriate name for the disease under consideration; Dr. Rigg's made no special claims, or discoveries, and had no idea of pre-empting it; his treatment was only an extension of what had already been done. Pyorrhea Alveolaris, on the other hand, was stilted, and had no special application, meaning only "a flow of pus from the alveolus."

The old term "conjoined suppuration of the gums and alveolus" recalled by Dr. Thackston, was perhaps as good as any. This is not a specific disease, but has many phases of manifestation. There is the peculiar separation of the gums from the roots, the flow of pus, the pockets, the channels down the roots of the teeth, sometimes penetrating very deeply, sometimes only superficial, and not even present in some cases. More has been said than the subject deserves as to whether it is local or constitutional; there are undoubtedly systemic conditions which favor its occurrence and continuance, but it is yet undecided what that condition is—whoever says *always* should study this up. There may be some poison in the system which favors the development of this disease under local stimulus, or local irritants. It is not due solely to systemic conditions independent of local causes; the two operate together.

Riggs said there was always the local irritant, and he was remarkably successful in his treatment, rarely ever failing, and yet rarely employing systemic treatment. What are the local irritants? Vitiated saliva, the accumulation of debris of food, epithelial scales, salivary calculus; all these may be, and often are sources of irritation. Serumal calculus is another irritant exciting and continuing the disease, and there may be disease of the margin of the alveolus in some form, causing disintegration or necrosis of the margin; the dissolved edges are left rough and continue the irritation. Organisms also here as elsewhere, play an active part, though the results of the presence of these organisms is not yet distinctly defined. There is room here for experiment and investigation. Nobody has yet brought out the tangible point on which to rest.

They are not found in every case. In some of the most severe cases organisms are not found in the debris. Yet the use of a germicide is entirely admissable and should be resorted to. When we know what we are dealing with, we know what is needed.

Different treatment is required for different phases of the disease, and it is important to differentiate.

Some features of treatment are common to all cases. There should first be local purification of the mouth and teeth, and the thorough removal of all deposits; going down deep into the pockets with explorers, all irritants must be found and removed with appropriate instruments (a large share of those sold for this purpose being entirely unfit for use). All roughened, ragged points of the alveolar margin must be burred off, and then sulphuric acid applied, as mentioned; it will act only upon tissues that are in a dying condition. Healthy tissues must be present to produce healthy exudations for healthy granulation. Granulations cannot attach where there are any deposits on the root. In treatment we should always know what we are aiming at; what it is we desire to do, and select the remedy that will accomplish that object. There is too much impirical use of therapeutic agents; but little medication is needed in any case; Riggs used none in many cases—sometimes a little tannin or carbolic acid, but never heroic treatment. Iodine in creosote Dr. Atkinson called his *big nigger*; he now uses a mild solution of salycilic acid, relying more on the surgical operation. We often fail from over therapeutic treatment. We should study causes and systemic peculiarities, and rely more on surgical treatment and the removal of debris. We have sloughing, half dead tissues instead of a healthy socket; even the gums should be scraped on the inner surface as the exudations are irritating. Peroxide of hydrogen is a grand agent. It prevents decomposition of debris, and is a germicide. The surfaces will heal, but I don't say there will always be restoration of tissues or process. The teeth, if loose, must be tied together as if loose, healthy granulation cannot take place—pabulum is thrown out, but the granulations are continually broken up. Clear away all irritants and treat with peroxide of hydragen, or salycilic acid in alcohol.

*Dr. Thackston* desired with hearty sincerity to thank his friend for this able address. When in the domain of *theory* it is easy to map out forces, causes, results, but in the domain of facts it is different. Our friend, in his clear and happy talk has laid great stress on local irritants, but where does all this local debris come from? The disease occurs with individuals who take the most scrupulous care, and resort to every expedient to secure healthy gums and teeth. A professional brother whose reputation is well established for great manipulative skill, employed every resource to check this disease. He resorted to every expedient suggested, but still the acrid, dissolving exudation continued until all the teeth were lost. Ordinary salivary calculus, having its origin in the secretions of the mouth, can be kept off, but there is another source—perhaps uric acid, as suggested, which resists all remedial agents. The teeth drop out, and the deposits are found—not at the neck, or on the sides of the root, but the very apex is enveloped in reddish-brown deposits, not from the saliva, and not from the gums, which are firmly attached, but from the blood itself; we can't get at it, and the tooth is lost.

*Dr. Winkler.* Do you infer that the deposit at the apex takes place prior to the loosening of the gum?

*Dr. Thackston.* I do, sir; it is not a salivary deposit.

*The President.* Sanguinary?

*Dr. Thackston.* Thanks. I could not find the word. The mouth is not the only place where calculius is found. We find it in the liver.

*Dr. Winkler.* It is not from the blood, it is from ducts.

*Dr. Thackston.* I have in my cabinet a tooth with incrustations of reddish-brown calculus at the apex of the root, which was not from the gums, but from the blood, it was systemic, and would eventually cause the loss of all the teeth.

*Dr. Teague.* Was it impossible to discover these deposits on the apex from without? Could an instrument not have been run down to the deposits?

*Dr. Thackston.* No sir; the tooth was firm in the socket, and the gums well attached.

*President Wardlaw* (calling Dr. Catching to the chair) said that he had seen cases of these dark sanguinary deposits on the roots when there was no detachment of the gums.

*Prof. Taft* said that these were not ordinary, but extreme cases. He had seen similar ones, but never except where there had been alveolar abscess. Where there was no detachment of the gums, with calcific or serumal deposits upon the roots, these had always been the subjects of alveolar abscess, but these cases were exceptional and rare. What we need to comprehend was the every day cases with which we have to deal. There are some which we cannot explain, but they are the rare cases.

*Dr. Rembert* (Natchez) spoke of serumal calculus as a product of pus flow--degenerating plasm thrown out, with calcific precipitation, as from the saliva, being deposited from serum or decomposing blood exuding through the tissues.

*Dr. J. J. R. Patrick* said that chemical analysis had never shown any difference between sanguinary and salivary calculus; the difference lay in the mode of deposit. Saliva is secreted and thrown out; the blood vessels or capillaries have to be ruptured to discharge serum. Salivary calculus is a secretion; sanguinary calculus is a concretion.

Adjourned to 8 P. M., when the discussion was continued.

*Dr. Morgan* said that a fundamental principle in the treatment of any disease was the removal of the cause. The one certain remedy for this disease was the removal of the teeth, which never fails to cure. This suggests the question whether the cause may not lie in the teeth themselves? Some authors say that the tooth proper embraces only the enamel, dentine, and cementum; others include the pulps; others again include the membrane. Is the trouble located in the cementum or the pericementum?

Perhaps back of that, as it is hereditary to a large extent. One reason why it is probably located in the cementum, is the fact that if the cementum is removed, the root being made perfectly smooth and separated from the periosteum, the trouble is removed and the gums heal up. The pressure from irritating substances tends to produce absorption; nature takes that method to relieve herself of trouble, but when the pressure is relieved, vital action is again carried on. Whatever may be the etiology and pathology, we have in every case of irritation inflammation and solution of parts. The incrustations are sometimes not calcareous. But soft pasty matter gives the same trouble, causing a breaking up of tissue. This darkish tartar is the result of the decomposition of the tissues of the blood, lime-salts are carried in the blood, and in the breaking up of the blood tissues, the lime-salts have to be disposed of, and they crystalize about the roots of the teeth. The question was asked if they were ever found where there was no fistulous opening at that point? Yes; where there has been alveolar abscess crystalization takes place.

Almost every individual spoke of removing necrosed alveolar process, but in treating this disease for nearly forty years, I have not seen exceeding six cases where there was any necrosed bone. The bone is softened, and the loose lime-salts are carried off by emunctories—perhaps used in building up other portions of the system, though we don't know that. We find softened alveolar process, but not necrosis. The lime-salts are absorbed, which is a physiological process. Necrosed bone is pathological and cannot undergo a physiological process, living tissues are absorbed and carried off in the general circulation. The surgical operation of removal of deposits and debris should be thorough and complete at the first sitting; the soft tissues are then broken up, and blood poured out, forming a fibrous coagulum which should remain there; it is protoplasm, and should not be disturbed. The pockets will fill up, as the socket does when a tooth has been extracted, gentle friction upon the gums is advisable.

Periosteum or cementum is never reproduced—nature does not do that. The tissue that is formed is not normal; it is largely of the nature of scar tissue; it is of low vitality and takes on inflammation more slowly. It is of the nature of cartilage and holds the teeth firmly in place. There is always a little chronic irritation at the end of the root which normal tissue would not tolerate. There is nothing equal to carbolic acid for destroying the fungus cells in the pockets and stimulating to healthy granulation. For two years have used Robinson's Remedy (carbolic acid and caustic potash) with great success. A second application is rarely necessary if the deposits have been properly removed. The disease is the local expression of systemic disease, and is found in whole families, parents and children alike. If taken early, in children, it is easily arrested, if strict cleanliness and friction of the gums are kept up. If neglected, the constitution is often undermined by it; it lays the foundation of delicate health; the vital forces are reduced, and they succumb readily to other diseases to which death is naturally attributed. Diet is an important ally in the cure, that proscribed for scurvy (to which it is allied) being what is needed; feed on the fat of the land, but let it be digestible. When hereditary, treatment is only palliative; the hereditary tendency cannot be removed. In young subjects the deposits are of a granulated, cheesy character—semi-solid. Cotton saturated with carbolic acid, or iodine, placed under the gum, and left until expelled by nature, will sometimes entirely relieve the ichorous discharge, with the aid of systemic treatment—not, however, with a view to general health, which should be left to the physician.

*Dr. J. Hall Moore* said that the result of his observation pointed to a complication with constitutional catarrh, which local treatment could not reach. In some cases there are no deposits on the teeth, the roots being clean, bright, and polished, but all the other symptoms present; pus exuding; deep pockets; the alveolar border roughened; but no tartar inany form, and always complicated with catarrh. Until the latter is cured pyorrhea will always return. Subject passed.

*Dr. B. S. Byrnes* read a paper embodying some incidents of office practice, imbedded third molars; a bicuspid held down by a retained temporary first molar in an adult; a deposit of salivary calculus as large as a molar with its roots, removed from an inferior left central incisor, etc.

ADJOURNED TO 9 A. M.—THIRD DAY.

*Dr. Morgan* stated that resolutions of impeachment had been offered, on charges of unprofessional and ungentlemanly conduct of one of the members of the Association, and moved that the President appoint a committee to investigate the charges and report at the next annual meeting.

Drs. Thackston, J. Hall Moore, and G. H. Winkler were appointed a Committee of Investigation of Charges.

Resolutions of thanks were tendered the officers of Walkin's Institute; the Y. M. C. A.; Vanderbilt University; the Tennessee Historical Society, and the Nashville Art Association.

A committee was appointed to draft resolutions on the death of Drs. Holmes, Best, Jobson and Redman.

*Dr.* ——— (a physician present) stated that some time ago, finding his teeth stained from the use of tobacco, he went to a dentist to have them cleaned. The dentist, on examination, pronounced his teeth, with the exception of the tobacco stains, to be in perfect order, finely developod, and of perfect form. He advised him to abandon the use of tobacco, which he did for several months, when he found his teeth all getting loose, with pus exuding around them. He took up tobacco again, and his teeth became perfectly firm, and the gums sound. He wished to ask why? how? what? He had no treatment, except the first cleaning from stains.

No reply. Subject passed.

Report of Committee on CHEMISTRY.

*Dr. Theo. Johnston* read a paper entitled:

GOLD; COHESIVE AND NON-COHESIVE.

He thought that the principles of chemistry involved in the preparation of gold for dental purposes should be taught in every dental college, but it is a subject of which the general practitioner knows little or nothing. If we understand clearly why our gold is, or is not cohesive, we should have less trouble in handling it. He had for his own satisfaction made researches and obtained facts which satisfied him as to the principles involved. He had no desire to divulge secrets which had been kept for years by manufacturers, or to reduce the price of gold, their remuneration for their labor being little enough, but he thought it would be a benefit to the profession to know more about the preparation of gold. The term *cohesive*, as applied by the dentist, is a misnomer; the pieces of gold are really united by welding, not by cohesion. He reviewed in detail the great variety of properties possessed by gold, in which it surpasses all other metals claiming that absolute purity was essential for welding.

His paper was highly commended by Dr. Morgan, who said that the Southern Dental Association ought to cultivate a young man who could produce such a paper, though in fact compelled to differ with him on some points. He did not think that gold was the most ductile metal, or that absolute purity was essential for welding. Abbey's gold is alloyed with six per cent. silver, as indicated by its color. But we want pure gold, and he hoped we would get it. He did not think that latent heat was actually forced out by hammering; the heat evolved was the result of friction among the molecules, but there was as much latent heat present in gold hammered or solid, as in the looser forms.

*Dr. Marshall* asked what percent of platinum, gold would receive without impairing its cohesive properties?

*Dr. Morgan* replied that he did not know, as he had not experimented in that direction. He did not use that form for welding, but only for artificial dentures. One pennyweight to the ounce would stiffen it and make it harder; two pennyweights would make it springy, equal to steel if not annealed.

*Dr. Theo. Johnston* said that we can only see the effects of heat, not the heat itself. All matter is porous, the pores being occupied by heat, which is driven out by compression, as water is squeezed from the pores of a sponge. Heated steel expands, but hammering drives the heat out; iron can be hammered till red hot.

*Dr. Salomon* said (in reply to Dr. Marshall's question) that the addition of platinum to gold does not destroy its welding properties. It will take three, six, and even ten per cent. It makes it very hard, but three or six per cent. is easily worked and very cohesive.

*Dr. Winkler* said that in platinum gold glinders, the platinum is beaten out in connection with gold, and sweated to it, making it very cohesive, but with great resistance to wearing. No. 3 will break instruments, but has a most beautiful appearance, for front fillings, being scarcely preceptible even though one-third of a front tooth be built down with it.

*Dr. Morgan* replied that heat never occupies space. It is not an entity but only a mode of motion; it is eliminated, or produced by the motion of the molecules (as if you were to run down the street just now—the motion would get up an immense amount of heat among your own molecules).

*Dr. Winkler* said that if molten gold is stirred with a platinum wire, the molecules of one being smaller than of the other, they flow in between and are thus mixed. Chemistry passed.

Operative Dentistry, temporarily passed, was taken up again.

*Dr. Crenshaw* said that the discussion of his paper was cut off by the hour of adjournment, but some remarks had been made which he wished to answer.

Dr. McKellops had said that the most prominent men had abandoned the electric mallet. He knew that some men, prominent in New York City, had abandoned it because it was rather complicated, and too much for ordinary men, but it was well worth the time, and perhaps some annoyance required to keep it in order. It was advisable always to have two--one in use and one out for repairs and adjustment----that was his plan, and he had no further trouble, simply using them alternately.

Dr. McKellops had also said that the tooth socket was liable to be destroyed by rapid separation. He would admit that it was even possible to open the suture in small children, but judgment and sense must be used in this, as in other things. The use of separators occasions less pain than separating with rubber.

Dr. Winkler had said that soft gold was better for margins, etc. He had run the gauntlet of all the different methods, and had concluded to settle down and stick to one thing. Everything required can be accomplished with cohesive gold if properly handled, but he did not hope to make any converts or to convince any one. He had presented--not new principles, but the principles laid down by Atkinson, Webb, and others.

*Dr. Beech* thought it was a narrow view to hold to one thing always. Many teeth that cannot be saved with cohesive gold, can be saved with other materials.

*Dr. Crenshaw* said that because some men can't do a thing, is no sign that it can't be done. To make a perfect system, it is necessary to confine ourselves to few materials, in order to simplify and avoid confusion.

*Dr. Winkler* said that he used soft foil at cervical walls because it was a better preservative from recurring decay. The invariable use of cohesive gold, with never an exception, requires great manipulative skill, but much painful labor to the operator, and long suffering of the patient, is avoided by the occasional judicious use of soft gold.

*Dr. McKellops* said it was not necessary for him to reply in regard to the electric mallet; no one would say that he did not know how to take care of it. He had had the best ever made, and the best batteries, and the best girl to take care of them, who understood her business perfectly and kept them in perfect order; but he objected to the blow, which was too harsh and too severe. Of course they were a great nuisance too, but we kept suitable assistants to take care of that part of it. When he spoke of injuries by wedging, he did not refer to the use of Perry's Separators. Perry always had the best of everything; but he spoke of rapid wedging, and he had seen Perry himself use wedges instead of his own separators. With folded tape, gradually increased day by day, no soreness is created. Forcing far apart with separators is very painful.

I like Bonwill's instruments, but I don't like a man who condemns me because I won't give him a certificate. The beauty of Bonwill's instrument is that the blow is perfect. You will be converted in a minute if you once try his mallet, but you have to learn how to handle it. I didn't like it at first, I changed the springs myself, so as to be able to use it in either hand. The electric mallet makes 2000 blows, Bonwill's will make 3000 blows a minute. I did not abandon the electric mallet because it was a nuisance, though it is true it has to be always in Philadelphia unless you have proper assistants. For powerful jaws, platinum gold wears like steel, and tooth color can be matched well. I have built down all the front teeth of a very large man, a blond who shows his teeth very much, but you can't tell they are filled. You don't hear the children cry out. "See the man with gold teeth!" It works as easy as lead; use the rolled gold in three grades of color; it is beautiful, durable, and makes no display.

*Dr. Staples* said he came from Texas to take a back seat and listen and learn. He was satisfied that twenty-four out of every twenty-five practitioners ought to use soft foil. Why? Because lack of thoroughness was the source of failure. Any man who had tried both would acknowledge that it was more difficult to use cohesive gold; it takes more skill, and more thoroughness. A man who is not thorough has no business using cohesive gold. Work should be done thoroughly and conscientiously even if you only expect to get twenty-five cents for it. Never get out of patience with the length of the operation, and don't stop till it is thoroughly finished. Am a hard foil man myself, have not bought five ounces soft foil in twenty years.

*Dr. Marshall* (Little Rock) said that he used both foils. Failures are due to the operator; to the power behind the plugger, not to the gold.

*Dr. Freeman* (Nashville) did not believe the dark line was always due to the other end of the plugger. It was unprofitable and a loss of time to talk about cohesive and non-cohesive gold. What we want is to know how to preserve teeth. Tin foil is sometimes put over an exposed pulp, as a cap, and it is good practice, but it may slip, and when the filling is finished appear as a dark line at the margin. The next operator will find that dark line at the cervical margin, but he will not find decay. Tin is compatible to dentos. Dr. Chase, of St. Louis, set forth that idea in 1869; was empirical to the extent of trying what he recommended, and found good results. Teeth that had previously failed at the cervical margins are saved by a tin lining. Tin hardens the tooth and preserves it, through the tubuli fill with coloring matter and discolor, but the tooth is preserved. I tell my patients beforehand to look for that, but that it don't mean decay.

*Dr. Louis Chisholm* (a retired veteran) said that required skill not attained by one in one hundred to perfectly protect cervical margins with cohesive gold. Retaining points should be made in solid bed-rock, and soft gold built right at the margin, across from one side to the other. If moisture cannot enter at the margins, the tooth is safe, no matter if there is a vacuum in the center.

*Dr. Crawford* thought the main point in the choice of gold was in accessibility. If the proper impact could be given, the tooth could be as pefectly filled with cohesive as with non-cohesive gold. We want minimum force for perfect condensation. The result rests with the hand and arm which wields the instrument. More will be accomplished by the hand than with mechanical appliance, and the test will be the length of time required for the operation, not the quantity of gold introduced, for if absolutely full the weight must be the same, for gold is gold, and weighs the same the world over. The cardinal thought in the paper read, was the establishment of a system, and the elevation of the standard by which it is controlled.

*Dr. Crenshaw*, author of the paper, closed the discussion. He thought it strange that Dr. McKellops had not found out that the blow of the electric mallet could be regulated, and at will, and adjusted to any degree of lightness wanted.

*Dr. Bonvill,* using his own mechanical mallet, had, in a clinic, created such inflammation and trouble in a tooth as to force its extraction, and yet the filling was very porous. This would not have been the case with the electric mallet. Subject passed.

A paper on Hygeine. REMARKS ON FOODS IN GESTATION AND LACTATION, by Dr. Bissel (South Carolina) was received too late for reading, except by title; was referred to the Committee on Publication.

*Dr. Catching* related a remarkable case of dental development. A six months child, born in 1871, began teething at the age of six months, erupting a full set of very small teeth, which were all shed within three months. At eleven months she began teething again, and at fifteen months had a second full set of teeth. These also soon crumbled away like chalk. When two and a half years old she weighed ten pounds, and had a third set of teeth. She suffered continually from then, and they were all extracted before she was four years old. At seven years old she weighed thirty pounds, and had four new teeth of the fourth set, in the upper front jaw. These were mere shells and being only an annoyance, were chipped off with the finger nails. At eleven years of age she began cutting the fifth set, which she now has. At the present age of fifteen she is as stout and hearty as any child of her age.

Casts of the mouth were shown, and the case vouched for by Dr. Catching. The child was fed on cod-liver oil and lime exclusively for the first eight months.

Drs. Chisholm, Richards, and Wright were appointed a committee to take the initiatory steps towards the establishment of a museum for the Southern Dental Association.

The business of the Association having been thus brought to a close, the usual resolutions of thanks were offered, the gavel was presented to the President and elections held.

Old Point Comfort, Va., was selected as the next place of meeting, the time to be fixed by the Executive Committee.

Dr. W. H. H. Thackston, Farmville, Va., was elected President.

Dr. B. H. Catching, Atlanta, Ga., First Vice-President.

Dr. J. R. Knapp, New Orleans, Second Vice-President.

Dr. W. H. Richards, Knoxville, Tenn., Third Vice-President.

Dr. L. B. Dotterer, South Carolina, Recording Secretary.

Dr. I. T. Crawford, Nashville, Tenn., Corresponding Secretary.

Dr. H. A. Lowrance, Athens, Ga., Treasurer.

Adjourned.

FRIDAY, 2 O'CLOCK P. M.

A letter from Dr. R. Finley Hunt, asking the appointment of a Committee of Conference, to meet a like committee from the National Dental Association, was laid on the table without action.

After the installation of the officers elect, the Association adjourned to meet on Monday, at the office of Dr. Morgan for clinics, after which adjourned *sine die*.

